# LIFESPAN SOCIOECONOMIC STATUS AND MORTALITY USING 1900–1940 CENSUSES: FINDINGS FROM THE NORMATIVE AGING STUDY

**DOI:** 10.1093/geroni/igae098.2074

**Published:** 2024-12-31

**Authors:** Victoria Marino, Joseph Ferrie, Ashley N Dorame, Eileen Graham, Avron Spiro III, Daniel Mroczek, Lewina Lee

**Affiliations:** National Center for Posttraumatic Stress Disorder, VA Boston Healthcare System, Boston, Massachusetts, United States; Northwestern University, Evanston, Illinois, United States; Boston University School Of Medicine, Boston, Massachusetts, United States; Northwestern University, Evanston, Illinois, United States; VA Boston Healthcare System, Boston, Massachusetts, United States; Northwestern University, Evanston, Illinois, United States; Boston University Chobanian & Avedisian School Of Medicine, Boston, Massachusetts, United States

## Abstract

Early and midlife socioeconomic status (SES) are linked to all-cause mortality, but few studies have examined their independent and joint associations using multidimensional, prospective SES measures and track mortality over extended periods. As described in the Dorame et al. presentation in this symposium, the Boston Early Adversity and Mortality Study (BEAMS) acquired prospective data on the early-life circumstances of three well-characterized male cohorts via linkage to the 1900-1940 US Federal Censuses. This study leverages BEAMS data to test the independent and joint associations of early and midlife SES with all-cause mortality in 1,303 men from a BEAMS cohort, the Normative Aging Study. Early SES, assessed at baseline (earliest available Census before age 18), was a composite combining paternal literacy (1=yes, 0=no) and z-standardized paternal occupational income. Midlife SES was a z-score composite of men’s occupational standing, education, and income at study entry (mean age=42). Mortality status was followed through December 2020 (mean follow-up since baseline: 77 years). Early SES was weakly and positively correlated with midlife SES (r=.17, p<.0001). In Cox regression, each 1-SD higher early SES was associated with 8% lower mortality risk (95%CI: 0.84, 1.00). Each 1-SD higher midlife SES was associated with 3% lower mortality risk (95%CI: 0.95,0.99), adjusted for early SES. The main effect of midlife SES weakened the association of early SES with mortality by only 2%. Early SES did not moderate the association of midlife SES with mortality. Our findings support largely independent contributions of early and midlife SES to all-cause mortality risk.

